# Correct Information Units in Narrative Discourse as an Indicator of Dementia

**DOI:** 10.1002/alz70857_107751

**Published:** 2025-12-25

**Authors:** Aparna Lalana Venugopal, Vandana V.P, Subasree Ramakrishnan, Faheem Arshad, Suvarna Alladi

**Affiliations:** ^1^ National Institute of Mental Health and Neurosciences, Bengaluru, Karnataka, India; ^2^ National Institute of Mental Health and Neurosciences, Bengaluru, India; ^3^ National Institute of Mental Health and Neurosciences [NIMHANS], Bengaluru, Karnataka, India

## Abstract

**Background:**

Correct Information Units (CIUs) are proven quantitative measures to analyze semanticity in the discourse of adults with neurogenic communication disorders, including dementia. Moreover, semantic impairment is often seen in the progressing stages of dementia, even before syntactic and phonological changes, in the majority of subtypes of dementia. The aim of the current study was to identify dementia from language samples elicited with a picture description task using locally validated picture stimuli and to understand the utility of CIUs and derivative measures to distinguish dementia from controls.

**Method:**

The study participants included age, gender, and education‐matched mild dementia individuals (*n* = 27) and controls (*n* = 30). The diagnosis of dementia and subtype was carried out by an expert neurologist in consensus with DSM‐IV criteria and standard criteria for AD, FTD syndromes and others. ACE‐III and CDR were administered to determine the general cognitive function and severity of cognitive decline in all the participants. The picture‐naming test and the semantic fluency test were done as additional measures of language. Language samples were elicited using a village scene, a new picture description stimulus. All the recorded language samples were transcribed and analyzed for semantic measures such as CIUs, CIU (%), and efficiency (CIUs per minute) based on the rule‐based scoring system. Diversity measures such as the number of total words, the number of different words, and the type token ratio (TTR) were obtained using Systematic Analysis of Language Transcripts (SALT) software. Further, we investigated the group differences in CIU and other related measures. Correlations of CIU and other related measures with standard language measures and cognitive scores were examined.

**Result:**

Individuals with dementia demonstrated lower efficiency in conveying information than controls. Additionally, the performance of individuals with dementia on CIU‐related measures (CIU total, CIU%, Efficiency) and the TTR was poor compared to controls. Furthermore, there was a high correlation between CIU measures (measures and overall cognition, naming, and semantic fluency.

**Conclusion:**

CIUs and efficiency measures using a simple picture description task can be used to discriminate dementia from neurotypical controls as they show poor efficiency and quantity in conveying correct information.

